# Oral Contrast-enhanced Ultrasonography Diagnosis of Pharyngoesophageal Diverticulum Resembling Thyroid Nodules or Lymph Nodes: Case Series

**DOI:** 10.1055/a-2525-5961

**Published:** 2025-04-14

**Authors:** Wanbing Qiu, Qianyi Dou, Lu-Yao Zhou, Jia Luo, Fushun Pan, Wei Wang, Yan-ling Zheng, Xiao-Yan Xie, Jinyu Liang

**Affiliations:** 1 71068 Department of Medical Ultrasonics, The First Affiliated Hospital of Sun Yat-sen University, Guangzhou, China

## Abbreviations

PD: pharyngoesophageal diverticulum

TN: thyroid nodule

FNA: fine-needle aspiration

US: ultrasound

IC-CEU: Sintracavitary contrastenhanced ultrasound

UCAs: ultrasound contrast agents

CEUS: contrast-enhanced ultrasonography

## Introduction

Pharyngoesophageal diverticulum (PD) is a rare disease characterized by an alimentary tract pouch or a circular dilated cavity arising from the esophagus, occurring mostly in the elderly (Herbella FA et al. Langenbecks Arch Surg 2012; 397: 29–35, Nehring P et al. Prz Gastroenterol 2013; 8: 284–289L). PD is incidentally detected and usually mimics a thyroid nodule (TN) (Pang JC et al. J Clin Ultrasound 2009; 37: 528–30, Singaporewalla RM et al. Head Neck 2011; 33: 1800–1803). Unnecessary invasive procedures, such as fine-needle aspiration (FNA) or surgery, are sometimes carried out (Cao L et al. Oncol Lett 2016; 12: 2742–2745, Ye-huan L et al. World J Surg Oncol 2015; 13: 131).

Ultrasound (US) is a first-line imaging modality for the diagnosis of TNs and has been widely used to predict malignancy risk of TNs (Ha EJ et al. Korean J Radiol 2018; 19: 623–631). This modality is aptly suited to visualize and characterize TNs in a safe, comfortable, and efficient manner, without exposing patients to radiation (Escalante DA et al. Surg Clin North Am 2022; 102: 285–307), but the accuracy of diagnosis is not satisfactory for PD (Salimi F et al. Ann Med Surg (Lond) 2020; 60: 515–517). Barium swallow pharyngoesophagogram is the primary diagnostic tool for PD, but it exposes patients to radiation (Yun PJ et al. J Thorac Dis 2017; 9: E787–E7891).

Intracavitary contrast-enhanced ultrasonography (IC-CEUS) is performed by injecting ultrasound contrast agents (UCAs) into sonographically accessible physiological or pathological body cavities to assess the morphology of the cavity and potential communication with adjacent structures or organs. Clinical applications of IC-CEUS include the gastrointestinal tract, abscesses, and fistulas (Kljucevsek D et al. Pediatr Radiol 2020; 50: 596–606, Mao R et al. J Crohn’s Colitis 2019; 13: 593–599). Oral contrast-enhanced ultrasonography (CEUS) is an application of IC-CEUS and is an effective method for diagnosing PD and avoiding unnecessary surgical operations. We have summarized the ultrasound characteristics of PD on US, oral CEUS, and intravenous CEUS and will present two typical cases in this case series.

## Case series

This case series includes 15 patients with PD diagnosed by oral CEUS between January 2019 and January 2023. Surgery or FNA was not performed on all of the PDs. Patients were examined bilaterally on both the axial and longitudinal planes of the thyroid using a US machine. Intravenous CEUS was performed using the contrast pulse sequencing (CPS) ultrasound imaging mode. Intravenous access was established via the elbow vein. Subsequently, 2.4 mL UCA were injected as an intravenous bolus per subject through the elbow vein, followed by a 5 mL 0.9% saline flush. Oral CEUS was performed by patients swallowing diluted UCA (5–10 mL SonoVue in 50 mL water, Bracco SpA, Milan) several times.

All lesions were located in the posterior aspect of the thyroid, 14 in the left lobe and one in the right. Clinical data for patients and sonographic characteristics for PD are described in Table 1 .

**Table:** **Table 1** Clinical data and sonographic characteristics for patients.

N0.	Age/sex	Chief complaint	Size (cm)	Location	Sonographic findings	Contrast mode	Oral CEUS characterization	Intravenous CEUS characterization
1	44/F	Asymptomatic, finding left neck mass	1.2	Left lobe	Heterogeneous hypoechoic lesion with clear boundary, multiple punctate strong echoes and anterior arc-shaped hyperechoic area. The lesion seemed connected with the esophagus	Oral	Positive	No-enhancement
2	48/M	Asymptomatic, finding suspected left neck mass and lymph node	0.6	Left lobe	Hypoechoic mass with clear boundary and hyperechoic foci moving with swallow	Oral	Positive	
3	32/M	Asymptomatic, finding suspected left neck mass	0.6	Left lobe	Hypoechoic mass with clear boundary and hyperechoic foci	Intravenous +Oral	Positive	No enhancement
4	43/M	Asymptomatic, finding left neck mass	0.9	Left lobe	Hypoechoic mass with clear boundary and strong echoes	Intravenous +Oral	Positive	No enhancement
5	34/F	Asymptomatic, finding left neck mass	1.7	Left lobe	Mixed echoic nodule with unclear boundary and hyperechoic foci associated with sign of air	Intravenous +Oral	Positive	Internal iso-enhancement and boundary ring enhancement
6	37/F	Asymptomatic, finding suspected left neck mass	1.1	Left lobe	Mixed echoic nodule with boundary hypoechoic rim	Intravenous +Oral	Positive	No enhancement
7	47/M	Asymptomatic, finding suspected left neck mass	1.6	Left lobe	Mixed echoic nodule with hypoechoic rim and internal hyperechoic foci associated with sign of air	Intravenous +Oral	Positive	Internal no enhancement and boundary ring enhancement
8	48/F	Pharyngeal foreign body sensation, secondary hyperparathyroidism	3.5	Left lobe	Mixed echoic nodule with boundary hypoechoic rim	Intravenous +Oral	Positive	Internal no enhancement and boundary ring enhancement
9	41/F	Asymptomatic, finding suspected right neck mass	1.2	Right lobe	Hyperechoic mass with hypoechoic rim and punctate strong echoes. The mass seemed connected with the esophagus	Oral	Positive	
10	33/F	Asymptomatic, Hashimoto's thyroiditis	0.4	Left lobe	Hyperechoic mass with arc-shaped hyperechoic areas. The hyperechoic area was associated with a comet-tail artifact and moved during swallowing	Oral	Positive	
11	13/F	Pharyngeal foreign body sensation, finding suspected left neck mass	1.1	Left lobe	Isoechoic mass with unclear boundary and internal multiple punctate strong echoes	Intravenous +Oral	Positive	No enhancement
12	33/M	Asymptomatic, finding left neck mass	1.3	Left lobe	Isoechoic mass with unclear boundary and internal punctate strong echoes	Oral	Positive	
13	50/F	Asymptomatic, finding left neck mass	1.3	Left lobe	Isoechoic mass with clear boundary and internal multiple punctate strong echoes	Intravenous +Oral	Positive	No enhancement
14	66/M	After thyroidectomy, finding suspected left neck mass	1.4	Left lobe	A central anechoic mass with boundary hypoechoic rim, internal punctuate hyperechoic foci with a comet-tail artifact. The mass seemed to move with the esophagus during swallowing	Intravenous +Oral	Positive	Internal no enhancement and boundary ring enhancement
15	30/M	Asymptomatic, finding left neck mass	0.8	Left lobe	Anechoic mass with clear boundary and internal macrocalcifications	Oral	Positive	

F: female; M: male; CEUS: contrast-enhanced ultrasonography; Positive: UCA perfused into lesion during swallowing.

**Fig. 1:**
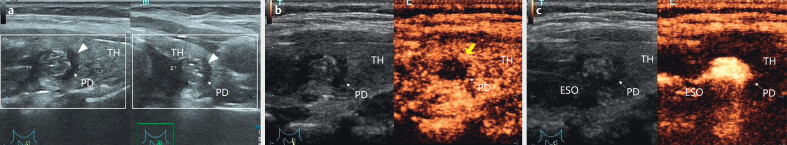
Images of a 13-year-old girl with suspected malignant neck mass: ( **a** ) Longitudinal sonogram and transverse sonogram revealed isoechoic mass with a peripheral hypoechoic rim (white triangle arrow) and internal punctuate echogenic foci. (PD: pharyngoesophageal diverticulum; TH: thyroid). ( **b** ) Intravenous CEUS revealed that the lesion showed no internal enhancement and boundary ring enhancement (yellow bold arrow). (PD: pharyngoesophageal diverticulum; TH: thyroid). ( **c** ) Oral CEUS revealed the lesion was significantly enhanced and connected to the esophagus after swallowing the UCA. (PD: pharyngoesophageal diverticulum; TH: thyroid; ESO: esophagus).

Four aspects of conventional ultrasonic features associated with PD were summarized as follows: (1) Echogenicity: PD manifested in various forms of the interior echoes, including hypoechoic, hyperechoic, isoechoic, anechoic, and mixed echogenicity; (2) Echogenic foci: Echogenic foci were usually present with various manifestations in PDs, including a hyperechoic area associated with the sign of air or a comet-tail artifact, macrocalcifications, and punctate echogenic foci ( Fig. 1 **a** , 2 **a** , 3 **a** ); (3) Margin: The PD boundary could be either clear or unclear. Some PDs displayed a hypoechoic rim at the boundary ( Fig. 1a ) or an anterior arc-shaped hyperechoic area; (4) Relationship with the esophagus: Some lesions seemed to have a connection with the esophagus. During swallowing, the movement of the echogenic foci correlated with the air movement within the esophagus. In general, the majority of PDs were heterogeneous, with strong internal echogenic foci and a hypoechoic rim at the boundary.

**Fig. 2:**
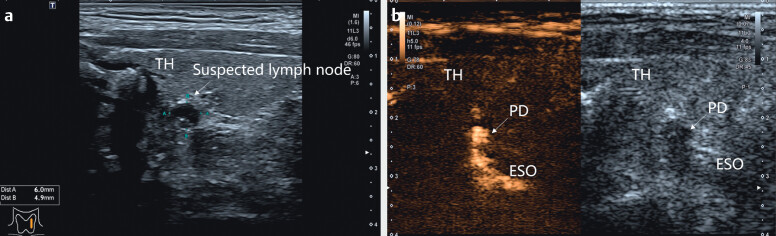
Images of a 48-year-old male with suspected thyroid nodule and lymph node: ( **a** ) Longitudinal sonogram revealed suspected lymph node located in the posterior of the thyroid with hypoechoic, clear boundary and hyperechoic foci. (TH: thyroid). ( **b** ) Oral CEUS revealed the suspected lymph node was enhanced and connected to the esophagus after swallowing the UCA. (PD: pharyngoesophageal diverticulum; TH: thyroid; ESO: esophagus).

**Fig. 3:**
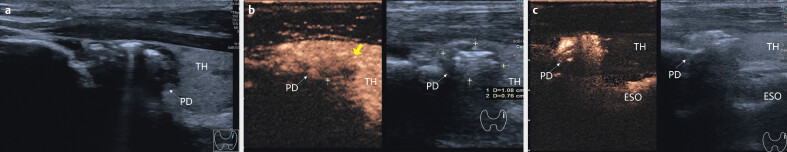
Images of a 34-year-old female with suspected thyroid nodule: ( **a** ) Transverse sonogram revealed mixed mass with unclear boundary and hyperechoic foci associated with a comet-tail artifact. (PD: pharyngoesophageal diverticulum; TH: thyroid). ( **b** ) Intravenous CEUS revealed the lesion with some UCA internal filling and boundary ring enhancement (yellow bold arrow). (PD: pharyngoesophageal diverticulum; TH: thyroid). ( **c** ) Oral CEUS revealed the lesion was enhanced and connected to the esophagus after swallowing the UCA. (PD: pharyngoesophageal diverticulum; TH: thyroid; ESO: esophagus).

All oral CEUS examinations were positive (the UCA passed rapidly through the esophagus and diffused into the lesion). The UCA was present in the interior of the lesion but not in the thyroid gland or lymph node ( Fig. 1 **c** , 2 **b** , 3 **c** ). Nine patients underwent intravenous CEUS. 8 lesions showed no enhancement, with 3 lesions being accompanied by peripheral rim enhancement ( Fig. 1b ). One lesion, characterized by a central hyperechoic area and hypoechoic rim, showed iso-enhancement and peripheral rim enhancement ( Fig. 3b ).

### Classic case 1

A 13-year-old girl underwent thyroid ultrasonography at another hospital due to a pharyngeal foreign body sensation. A mass suspected of being malignant was detected in the left lobe of the thyroid. Her parents were very worried. Subsequently, accompanied by her parents, she came to our hospital for further evaluation. To screen for thyroid cancer, she underwent thyroid US and CEUS examinations. The examinations were performed by an experienced radiologist who has 5–10 years of experience using the CEUS technique. The ultrasound sonograms revealed an isoechoic mass with punctuate echogenic foci and peripheral hypoechoic rim in the left lobe ( Fig. 1a ). The mass exhibited movement relative to the thyroid gland. However, no significant change was observed in the internal echogenicity of the mass when the girl was instructed to swallow. Intravenous CEUS revealed that the lesion showed no enhancement with peripheral ring enhancement ( Fig. 1b ). Therefore, the mass was suspected to be a PD misdiagnosed as TN. To prove this, oral CEUS was performed. It showed that the diluted UCA perfused into the lesion through the esophagus when the girl swallowed the UCA many times ( Fig. 1c ). It confirmed that the suspected malignant TN was actually PD. Ultimately, the little girl avoided unnecessary fine-needle aspiration or surgery, thereby sparing her parents additional anxiety.

### Classic case 2

A 48-year-old male was diagnosed with suspicion of malignant TN in the left lobe and suspected metastatic lymph nodes (internal punctate echogenic foci) in left neck region VI based on previous ultrasonography examination. An experienced radiologist conducted further investigations. During the observation of the suspicious lymph node ( Fig. 2a ), we found that the lymph node was closely related to the posterior esophagus and fluid flow appeared to be visible during swallowing. The patient was instructed to swallow diluted UCA several times. We observed that the UCA entered into the suspected lymph node through the esophagus ( Fig. 2b ), which confirmed the diagnosis of PD.

## Discussion

PD occurs in areas of muscular gap at the transition of the cricopharyngeal, inferior constrictor of the pharynx, and esophageal intrinsic muscles (Herbella FA et al. Langenbecks Arch Surg 2012; 397: 29–35). Clinical findings of PD include dysphagia, food regurgitation, compression of the esophagus in the case of a large diverticulum or it can be asymptomatic for years (Marcy PY et al. Thyroid 2010; 20: 1317–1318). In this case series, most patients were asymptomatic and went to our hospital with suspicion of a thyroid mass based on an examination.

PD is often misdiagnosed as a TN or a lymph node (classic case 2). It occurs in the posterior aspect of the thyroid. Sometimes it can be mistaken for a tumor on the dorsal side of the thyroid gland (Huang HJ et al. J Med Ultrason (2001) 2020; 47: 279–285, Wang Y et al. J Clin Ultrasound 2016; 44: 333–338). Most of the PD cases in our study (n=14) were located in the posterior aspect of the thyroid left lobe. This may be related to the anatomical structure, i. e., the esophagus is slightly curved to the left, and the thickness of the left esophageal myometrium is significantly smaller than that of the right myometrium (Fitchat NA et al. J Laryngol Otol 2019;133:515–9). The anterior arc-shaped hyperechoic area was seen in one case and the peripheral hypoechoic rim in 4 cases, which is characteristic of the esophageal wall (Cui XW et al. Ultrasound Med Biol 2015; 41: 975–981).

Barium radiography is the conventional main means of diagnosing PD. However, it may result in radioactive exposure of patients (Mantsopoulos K et al. Ultraschall Med 2017; 38: 377–394). Undoubtedly, using water or sparkling water to distinguish PD from TNs is a convenient and efficient method during ultrasound examination. We excluded PD patients who could be identified with these methods. In this case series, the patients we included showed no significant changes in lesions when swallowing saliva or water. Therefore, after conventional US, we instructed patients to swallow the diluted UCA several times. All oral CEUS examinations were positive. The entire process of the UCA rapidly passing through the esophagus and subsequently diffusing into the lesion could be observed by means of oral CEUS. The UCA was present in the interior of the lesion but not in the thyroid gland.

Since we did not observe any apparent connection between the lesion and the esophagus, and the clinical requirement was to screen for thyroid cancer, 9 patients also underwent intravenous CEUS. 8 cases showed no enhancement in the lesions, 3 cases also showed ring enhancement, which may be the esophageal wall enhancing, one lesion presenting multiple internal hyperechoic foci ( Fig. 3a ) showed iso-enhancement and boundary ring enhancement ( Fig. 3b ). The appearance of iso-enhancement may be caused by artifacts on CEUS. Highly echogenic interfaces, particularly at gas/soft tissue margins or regions of dense calcification, may not be completely subtracted and would appear on the contrast image and B-mode image (Fetzer DT et al. Abdom Radiol (NY) 2018; 43: 977–997).

The sign of air in the PD was the most important feature for differential diagnosis from TNs. However, the static air in the PD was hyperreflective and might mimic microcalcifications or psammoma bodies. PD may be misinterpreted as a malignant thyroid tumor, especially papillary thyroid carcinoma (Achille G et al. Endocr Metab Immune Disord Drug Targets 2019; 19: 95–99, Chen X et al. J Clin Ultrasound 2021; 49: 527–532). In addition, chronological changes in the internal echo resulting from probe compression or swallowing actions were also crucial for the differential diagnosis (Lixin J et al. Eur J Radiol 2011; 80: e13–e19). There were similar situations with our cases. These patients underwent intravenous CEUS because they were initially misdiagnosed with a suspicion of malignant TNs. During the examination, there was no obvious change in the lesions due to probe compression or swallowing. This caused us to question the initial diagnosis of PD. Therefore, no enhancement on intravenous CEUS and obvious enhancement on oral CEUS could result in a definitive and accurate diagnosis of PD.

The limitation of this case series is that it is based only on the patients’ chief complaints and imaging findings and there is no comparison with barium radiography or pathological results. In the follow-up medical treatment process, 14 patients did not undergo barium radiography or surgery, while one patient ( **Fig. S1, supplementary material** ) underwent surgery due to thyroid cancer.

## Conclusion

PD is a rare disease and usually mimics TNs. Patients are usually asymptomatic and occasionally present symptoms such as a pharyngeal foreign body sensation and dysphagia. If PD is misdiagnosed as a thyroid carcinoma, unnecessary invasive procedures may be performed. This case series reminds radiologists that they should consider the possibility of PD when detecting a suspected nodule located in the posterior aspect of the thyroid or LN. Oral CEUS is a noninvasive and radiation-free way to distinguish PD from TN.

## Patient consent for publication

Written informed consent was obtained from all patients prior to the publication of this case series.

## Supplementary Material

Supplementary Material Zusatzmaterial

