# Supplementary figures and images for: Oral Contrast-enhanced Ultrasonography Diagnosis of Pharyngoesophageal Diverticulum Resembling Thyroid Nodules or Lymph Nodes: Case Series
Source: Ultrasound Int Open. 2025 Apr 14;11:a25255961. doi: 10.1055/a-2525-5961 (PMC12039950; doi:10.1055/a-2525-5961)

Supplementary Figure

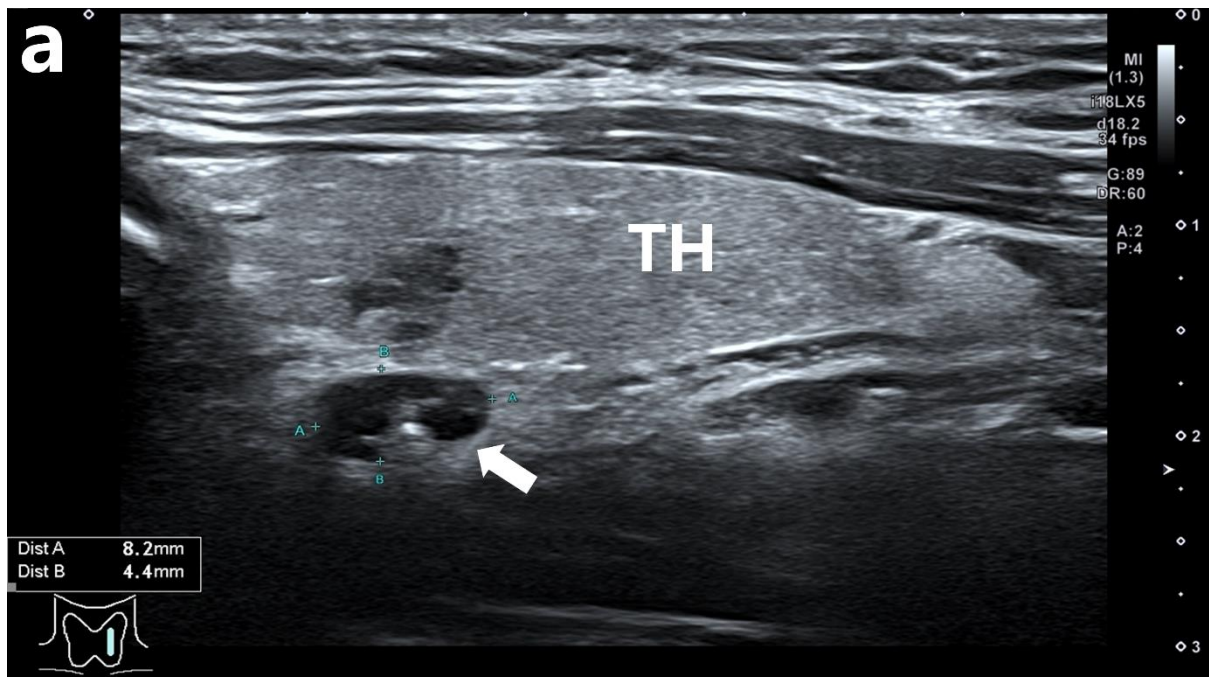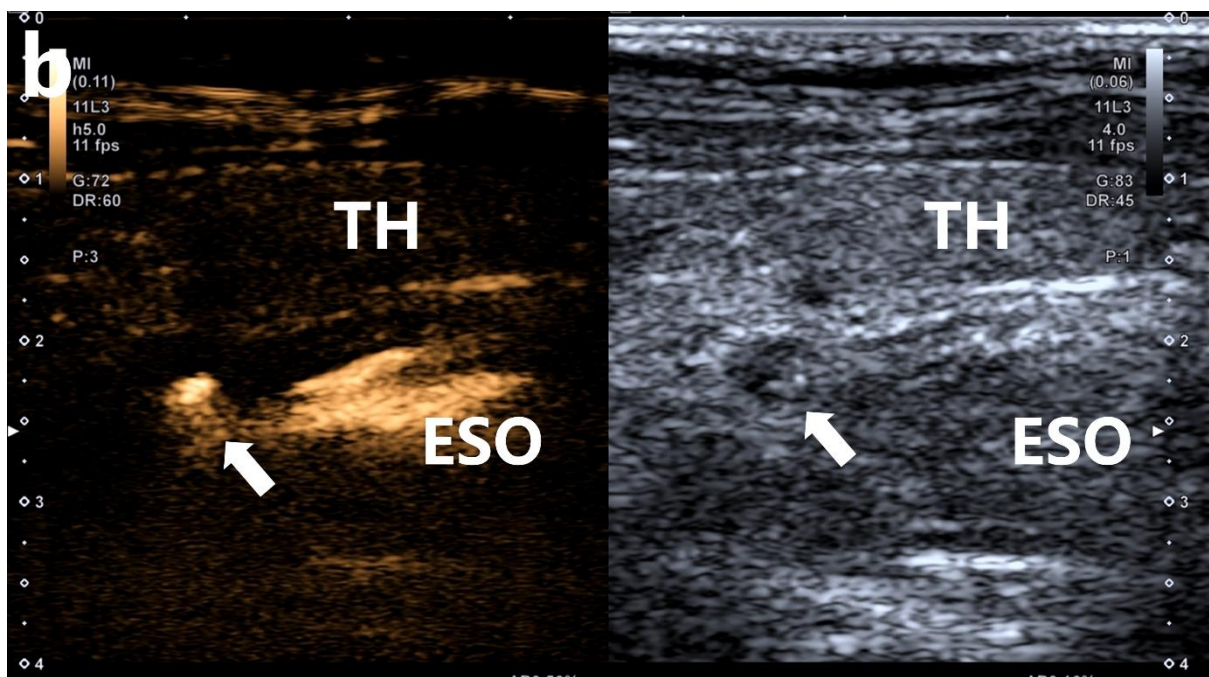

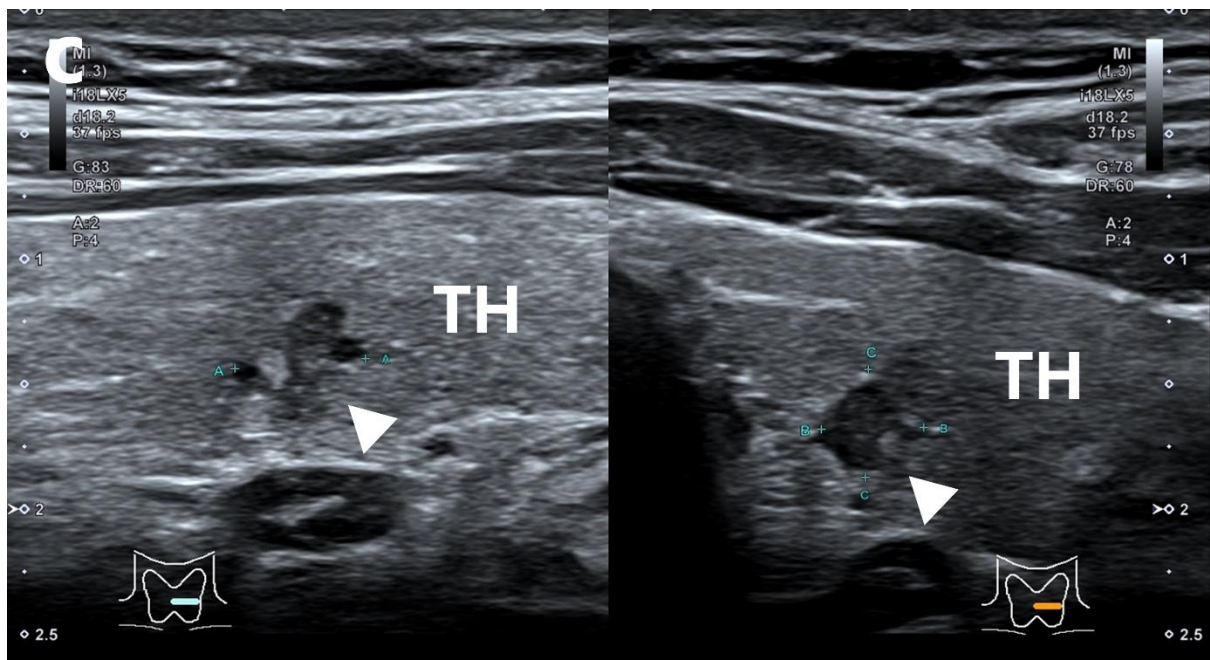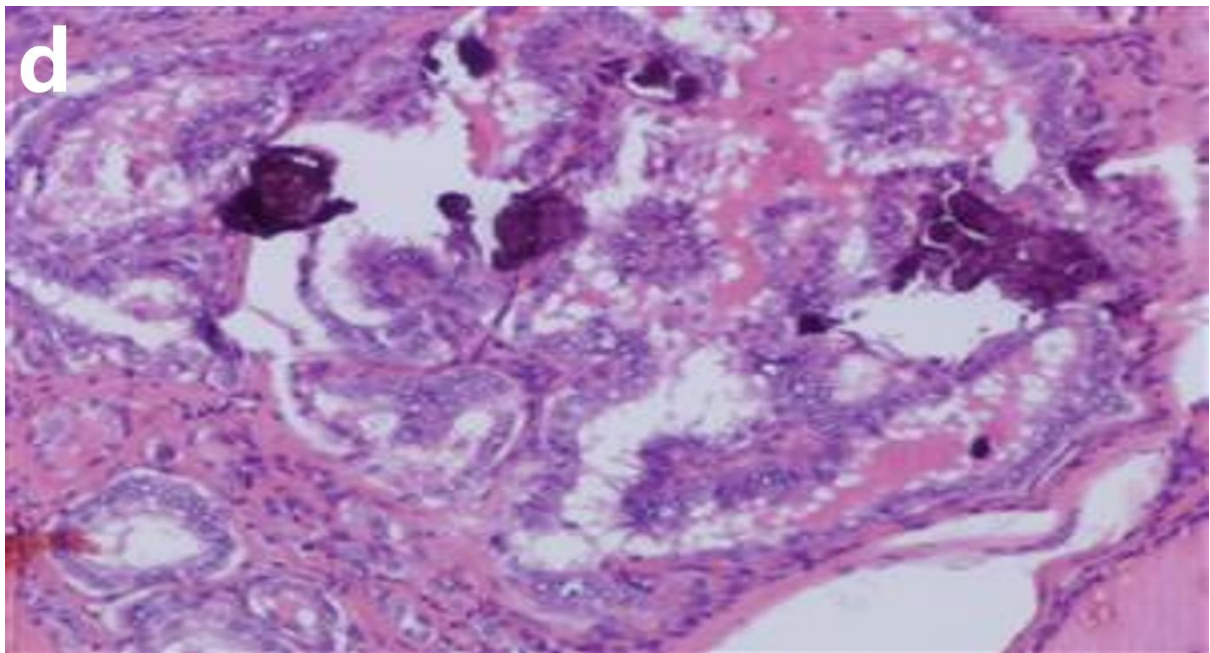

Supplement: Supplementary file 1 — Supplementary Material [file 10-1055-a-2525-5961_25295438.pdf]
